# Designing Practical Motivational Interviewing Training for Mental Health Practitioners Implementing Behavioral Lifestyle Interventions: Protocol for 3 Pilot Intervention Studies

**DOI:** 10.2196/44830

**Published:** 2023-03-16

**Authors:** Arlene Taylor Dalcin, Christina T Yuan, Gerald J Jerome, Stacy Goldsholl, Eva Minahan, Joseph Gennusa, Tyler Fink, Kimberly A Gudzune, Gail Lois Daumit, Faith Dickerson, David A Thompson, Nae-Yuh Wang, Steve Martino

**Affiliations:** 1 Division of General Internal Medicine Department of Medicine Johns Hopkins University School of Medicine Baltimore, MD United States; 2 Welch Center for Prevention, Epidemiology, and Clinical Research Johns Hopkins Medical Institution Baltimore, MD United States; 3 Department of Health Policy and Management Johns Hopkins University Bloomberg School of Public Health, Baltimore, MD United States; 4 College of Health Professions Towson University Towson, MD United States; 5 Department of Psychology Sheppard Pratt Baltimore, MD United States; 6 Department of Anesthesiology and Critical Care Medicine Johns Hopkins School of Medicine Baltimore, MD United States; 7 Department of Psychiatry Yale University West Haven, CT United States; 8 VA Connecticut Healthcare System West Haven, CT United States

**Keywords:** motivational interviewing, training, cardiovascular disease, lifestyle interventions, community mental health

## Abstract

**Background:**

Motivational interviewing (MI) is an evidence-based, patient-centered communication method shown to be effective in helping persons with serious mental illness (SMI) to improve health behaviors. In clinical trials where study staff conducted lifestyle interventions incorporating an MI approach, cardiovascular disease (CVD) risk profiles of participants with SMI showed improvement. Given the disproportionate burden of CVD in this population, practitioners who provide somatic and mental health care to persons with SMI are ideally positioned to deliver patient-centered CVD risk reduction interventions. However, the time for MI training (traditionally 16-24 hours), follow-up feedback, and the coaching required to develop and maintain patient-centered skills are significant barriers to incorporating MI when scaling up these evidence-based practices.

**Objective:**

We describe the design and development of the following 2 scalable MI training approaches for community mental health practitioners: real-time brief workshops and follow-up asynchronous avatar training. These approaches are being used in 3 different pilot implementation research projects that address weight loss, smoking cessation, and CVD risk reduction in people with SMI who are a part of ALACRITY Center, a research-to-practice translation center funded by the National Institute of Mental Health.

**Methods:**

Clinicians and staff in community mental health clinics across Maryland were trained to deliver 3 distinct evidence-based physical health lifestyle interventions using an MI approach to persons with SMI. The real-time brief MI workshop training for ACHIEVE-D weight loss coaches was 4 hours; IMPACT smoking cessation counselors received 2-hour workshops and prescribers received 1-hour workshops; and RHYTHM CVD risk reduction program staff received 4 hours of MI. All workshop trainings occurred over videoconference. The asynchronous avatar training includes 1 common didactic instructional module for the 3 projects and 1 conversation simulation unique to each study’s target behavior. Avatar training is accessible on a commercial website. We plan to assess practitioners’ attitudes and beliefs about MI and evaluate the impact of the 2 MI training approaches on their MI skills 3, 6, and 12 months after training using the MI Treatment Integrity 4.2.1 coding tool and the data generated by the avatar-automated scoring system.

**Results:**

The ALACRITY Center was funded in August 2018. We have implemented the MI training for 126 practitioners who are currently delivering the 3 implementation projects. We expect the studies to be complete in May 2023.

**Conclusions:**

This study will contribute to knowledge about the effect of brief real-time training augmented with avatar skills practice on clinician MI skills. If MI Treatment Integrity scoring shows it to be effective, brief videoconference trainings supplemented with avatar skills practice could be used to train busy community mental health practitioners to use an MI approach when implementing physical health interventions.

**International Registered Report Identifier (IRRID):**

DERR1-10.2196/44830

## Introduction

### Background

Addressing inequities in cardiovascular health is a critical public health objective, particularly for persons with serious mental illness (SMI) who experience alarmingly high rates of cardiovascular disease (CVD) risk factors (obesity, hypertension, dyslipidemia, and diabetes mellitus) and risk behaviors (tobacco smoking, physical inactivity, and unhealthy diet) [1-4]. Lifestyle interventions, especially those that adopt a patient-centered approach, have been shown to be effective in helping persons with SMI to improve a wide range of health behaviors, including weight loss management, smoking cessation, and diabetes management [5,6]. A foundational approach for many of these lifestyle interventions is motivational interviewing (MI), which is an evidence-based, patient-centered communication method aimed at evoking patients’ motivations for change [7]. MI practitioners adopt a collaborative and empathic attitude, empower patients to act as an equal partner in their disease management, and use specific and core patient-centered communication skills to guide them toward change.

Patients’ CVD risk profiles improved significantly in clinical trials in which the study staff incorporated an MI approach to conduct lifestyle interventions among participants with SMI [8-12]. To lessen the disparity of CVD risk experienced by this population on a wider scale, evidence-based programs to improve cardiovascular health need to be implemented in community mental health settings where patients routinely receive their care [13]. Real-world practitioners in these settings need the training not only to deliver the CVD health intervention itself (the requirements of which will likely differ based on education and prior experience) but also for the specific preparation necessary to incorporate an MI approach. Although some mental health practitioners may have had previous exposure to MI, the method involves a complex skill set such that attending a seminar or a traditional 2- to 3-day in-person workshop alone is usually insufficient to build competency [14-16]. A meta-analysis of MI training studies suggested that for learners to gain and sustain MI skills after a workshop training, an average of 3 to 4 coaching sessions with performance feedback from practice sessions over several months were needed [17]. Yet, when scaling up evidence-based practices (EBP) for implementation research, the stated amount of training to deliver patient-centered behavior change lifestyle interventions may be harder to accomplish among practitioners in community mental health settings who have demanding caseloads and limited time for additional training activities [18]. Furthermore, because of the diverse occupational backgrounds of the health care team in these real-world community settings (which often include physicians, nurses, therapists, and frontline day program staff) [19], it is essential to develop MI training that is both tailored and acceptable to a variety of practitioners.

There are a few promising formats to efficiently train busy mental health care practitioners in MI [20]. Synchronous or real-time trainings over videoconferencing can be used to provide learners with a basic introduction to MI. Live videoconferencing, although still requiring coordinating schedules of learners and trainers, can simulate an in-person event. Videoconference trainings may be shorter than standard multiday trainings and can be broken into smaller chunks of time; this may be more appealing to managers and allows learners time to meet their other work demands. This format also allows practitioners the opportunity to reflect on and integrate the concepts they are learning between training sessions.

Web-based self-study programs that include video and workbook activities can be entirely asynchronous or may integrate a *live component* in which learners meet with trainers during scheduled times [21-23]. Both real-time synchronous and web-based training options have the advantage of being able to reach a wider audience of practitioners, including those working in rural settings or where skilled trainers of MI are not available, than in-person events. Both approaches reduce the travel time and cost for trainers and limit the need for learners’ transportation, workshop space, and other expenses associated with in-person workshops. When implementing the intervention at multiple locations, videoconference training provides a practical way to reach several sites simultaneously. Constraints around social distancing imposed during the COVID-19 pandemic have created the need for effective options other than traditional in-person trainings for teaching different counseling approaches. Although these modalities have been used more frequently in the past few years, research on the impact of web-based [22] and video trainings in MI remains limited [24].

Another scalable remote training format for MI involves the use of computer simulations [20,25,26]. SIMmersion [27] offers video-interface trainings; videotaped role-play conversations with human actors give learners the ability to practice and receive coaching and feedback on their recorded responses [20,28]. Avatar-interface programs, by contrast, provide learners with a virtual reality training experience to learn and practice their skills with simulated avatar patients [29,30]. Avatar trainings may involve a human trainer [31] or consist of fully automated, preprogrammed simulations; some platforms also offer coaching and feedback features to learners. Kognito [32] offers several avatar simulations in which the users enter an interactive virtual environment and engage in role-play conversations with emotionally responsive virtual humans on a variety of topics in health care, mental health, and patient-provider communication. Their approach combines the science of learning, the art of conversation, and the power of gaming technology. Avatar-interface programs can be used as stand-alone or as follow-up MI practice opportunities; they have the advantage of allowing learners to practice their skills in a convenient and private environment.

### Objective

Despite the many different approaches to training and the demonstrated effectiveness of MI [6], the amount and type of training necessary for real-world practitioners to develop and sustain proficiency in the method is uncertain [24]. In the context of 3 pilot implementation research projects that address weight loss, smoking cessation, and CVD risk reduction among persons with SMI, we aimed to develop scalable training in MI. This paper will provide a brief description of the 3 pilot projects; their corresponding setting and intervention; the design and development of the 2 MI training approaches, real-time brief workshops and follow-up asynchronous avatar training used to implement the EBP; and how we propose to evaluate the impact of the training on practitioners’ skills, attitudes, and beliefs about MI.

## Methods

### Implementation Pilot Studies Overview

The Johns Hopkins ALACRITY Center for Health and Longevity in Mental Illness is a research-to-practice translation center funded by the National Institute of Mental Health that aims to reduce premature mortality from CVD in people with SMI [33,34]. There is an untapped opportunity to develop scalable and generalizable solutions that integrate physical health care into mental health service sectors, which have not historically provided such services. Through the ALACRITY Center, we are conducting 3 pilot implementation projects in community mental health settings to study strategies for the adoption and integration of evidence-based CVD risk reduction interventions related to (1) weight loss; (2) tobacco smoking cessation treatment; and (3) hypertension, dyslipidemia, and diabetes care for people with SMI. This study is a substudy within the ALACRITY Center methods core and focuses on the 2 types of innovative MI training: real-time brief workshop training over videoconferencing and asynchronous avatar training, which span the 3 projects described in the following paragraphs.

ACHIEVE-D (Adapting an Evidenced-based Weight Management Intervention and Testing Strategies to Increase Implementation in Community Mental Health Programs) is a 6-month, 2-arm, weight loss pilot cluster randomized controlled trial (RCT), which is designed to assess the degree to which the implementation strategy, that is, performance coaching, impacts psychiatric rehabilitation program (PRP) coaches’ knowledge, self-efficacy, and fidelity to the delivery of the adapted ACHIEVE-D weight loss program curriculum when compared with a standard control group. ACHIEVE-D is a behavioral weight loss program designed for persons with SMI that provides basic weight loss health education using an MI approach, which was adapted from an intervention tested in a previous RCT [10,12,35]. Coaches in PRPs will be provided with the materials, trained on all aspects of the ACHIEVE-D weight loss curriculum, and encouraged to use MI skills in their interactions with the participants.

IMPACT (Promoting Evidenced-Based Tobacco Smoking Cessation Treatment in Community Mental Health Clinics) is a pretest-posttest design–based 12-month observational study that aims to examine the effects of an implementation intervention on mental health providers’ delivery of four EBP related to smoking cessation treatment for clients with SMI: (1) assessment of smoking status, (2) assessment of willingness to quit, (3) behavioral counseling, and (4) pharmacotherapy prescribing. Psychiatrists and mental health counselors at 5 community mental health clinics will receive training to implement smoking cessation practices, incorporating an MI style in their interactions with the patients. Counselors and prescribers will be encouraged to use MI to understand their patients’ smoking behavior and engage them in smoking cessation treatment.

RHYTHM (A Quality Improvement Process to Support Delivery of Cardiovascular Care in Community Mental Health Organizations) is a pretest-posttest design–based 12-month pilot study examining the effects of an adapted 5-step Comprehensive Unit–Based Safety Program implemented during care coordination aimed at reducing CVD risk for patients participating in behavioral health home programs [36]. Behavioral health home staff will receive the training necessary to implement the program, which will include the use of MI as a way of interacting with patients as they discuss various aspects of their patients’ CVD care.

### Participants Receiving MI Training

In these pilot implementation projects, we are assessing the use of MI among clinician and staff participants who have been trained to deliver the lifestyle interventions to mental health clients. Practitioners recruited from community mental health settings across Maryland will implement the 3 projects. Only English-speaking adults (aged ≥18 years) who provide informed consent are enrolled. Additional inclusion criteria include willingness to provide baseline data, attend training, implement the study intervention, and participate in follow-up data collection. Exclusion criteria across the 3 projects are minimal and exclude any staff who express an intention to leave the facility before project completion. ACHIEVE-D enrolled interested residential staff and employees at the PRP program as coaches. IMPACT participants include community mental health clinic staff (eg, licensed counselors, psychologists, and social workers) and prescribers (eg, nurse practitioners and psychiatrists). RHYTHM participants include PRP staff and health home nurses who are currently employed at the study sites.

### Ethics Approval

Approval to conduct the studies and the associated MI trainings was granted by the Johns Hopkins Institutional Review Board (IRB) protocols. ACHIEVE-D study received IRB approval in March 2021 (IRB00247344). IMPACT study received IRB approval in January 2021 (IRB00231836), and RHYTHM study received IRB approval in December 2020 (IRB00269855).

### MI Training

MI Training for the ALACRITY Center studies consists of 2 distinct approaches: real-time brief workshop training over videoconferencing and asynchronous avatar training.

#### Real-time Brief Workshop Training

##### Overview

Traditional introductory MI training is typically in person and is 16 to 24 hours long. However, based on the preferences expressed by stakeholders at study sites before initiating the projects, we present brief, real-time, and standard introductions to MI over videoconferencing uniquely tailored for each project. The MI trainer (ATD) is an MI practitioner, is a member of the Motivational Interviewing Network of Trainers (MINT), and has extensive experience in training practitioners who treat patients with SMI in the context of health services research. Following the guidelines recommended by MINT members [37], each training session invites the learners to consider why MI is useful in conversations around health behavior change and includes a definition of the method; a discussion of the MI spirit (ie, the accepting, compassionate, collaborative, and empowering style used in MI); an introduction to patient language related to change (change talk and sustain talk); and a brief overview of the following core MI skills: open-ended questions, affirmations, reflections, summaries, and collaborative information exchange [7]. Table 1 presents the real-time training content.

The amount of time devoted to each topic and opportunities for interaction vary by study. To increase the feasibility and acceptability of the workshops, the length of training for each project was negotiated in advance with the site leadership. ACHIEVE-D weight loss coaches will receive 4 hours of MI training. Psychiatrists prescribing smoking cessation pharmacotherapy in IMPACT will receive 1 hour of MI training; mental health counselors who provide the smoking cessation counseling will receive 2 hours of MI training. RHYTHM health home staff who implement the CVD risk reduction program will receive 4 hours of MI training.

**Table 1:** Real-time training content.

	Study and the behaviors targeted by participants			
	ACHIEVE-D—weight loss	IMPACT—smoking cessation		RHYTHM—CVD^a^ risk reduction
	PRP^b^ staff	Prescribers	Counselors and Nurses	Health home staff
Participants, n	21	35	51	20
Training length (hours)	4	1	2	4
Training agenda	Why use MI?^c,d^ What does not help?^d^ Righting reflex Ambivalence Language of changed What helps?^d^ MI spirit Good practice video: MI skills occur with spirit^d,e^ MI skills: OARS^d,e^ Reflections guide the conversationd Responding to discord Introduce collaborative information exchanged Providing feedback Using leaders guides MI is not like a recipe Learning MI Introduction to the avatar	Why use MI?^d^ Ambivalence and change talk^d^ What helps?^d^ How MI works: Good practice video^d^ Brief review of core MI skills Collaborative information exchanged What it takes to learn MI Introduction to the avatar	Why use MI?^d^ What does not help?^d^ Righting reflex Ambivalence Language of changed What helps?^d^ MI spirit Good practice video: MI skills occur with spirit^d^ MI skills: OARS^d^ Introduce collaborative information exchanged MI is not like a recipe Learning MI Introduction to the avatar	Why use MI?^d^ What does not help?^d^ Righting reflex Ambivalence Language of changed What helps?^d^ MI spirit Good practice video: MI skills occur with spirit^d^ MI skills: OARS^d^ Reflections guide the conversation^d^ Responding to discord Introduce collaborative information exchanged Providing feedback Using leaders guides MI is not like a recipe Learning MI Introduction to the avatar

^a^CVD: cardiovascular disease.

^b^PRP: psychiatric rehabilitation program.

^c^MI: motivational interviewing.

^d^Included an interactive component (eg, chat response, poll, brief discussion, and practice exercise).

^e^OARS: Open-ended questions, Affirmations, Reflections, Summaries

##### Development

Owing to the advent of the COVID-19 pandemic, all MI trainings were modified from an in-person format to delivery over videoconferencing. Although there is not much known about how best to adapt the content of in-person experiential training workshops for this purpose, MINT members communicated ideas for the transition on the e-forum of their listserve [38]. Additional ideas for modifying training activities were offered during workshops, and a monthly virtual trainers meeting for sharing activities, ideas, and successes is ongoing for any interested MINT member. As a result of participation in these various collaborative conversations, the first author (ATD) modified several training activities for the videoconferencing format. One key consideration was how best to engage participants and encourage them to try different components of the MI method while being observed by others on screen rather than in the privacy of small groups. The trainer incorporates multiple activities, alternating training techniques to keep learners interested, including the use of written transcripts with change talk and sustain talk indicated in different colored fonts and small breakout rooms for practice exercises [39,40]. Efforts are made to switch styles every 20 minutes to best engage learners. Anonymous polling provides low-stakes opportunities for learners to select the most MI-consistent practitioner strategy in response to a vignette (eg, choose an evocative question, select the reflective listening statement, or identify the best example of a collaborative information exchange); the chat function allows the trainees, who may be less comfortable or quick to respond, opportunities to pose questions or respond to trainer requests.

All synchronous, real-time trainings will be held over videoconference and scheduled at a time that accommodates most practitioners at each site. As it is challenging to synchronize participants from multiple sites where the research is being conducted, for anyone unable to attend the real-time meeting, recorded Zoom sessions (Zoom Video Communications) will be made available for viewing. The first author developed a tailored handout resource packet with specific examples of how to use MI when discussing each project’s target behavior change; this material will be provided to practitioners to further support their MI skill acquisition during and after training.

#### Asynchronous Avatar Training

##### Overview

The ALACRITY Center partnered with a web-based experiential learning company, Kognito [32], to develop the asynchronous avatar training. This development process with Kognito took approximately 14 months and resulted in the products described in the following sections. After the completion of their real-time MI training, the practitioners are given access to the web platform and are encouraged to register for an account that houses their study-specific avatar training and simulation. The first component is a 12-minute didactic module, “Motivational Interviewing Techniques*,”* which uses an avatar coach to provide an overview of the method. This module is identical for each of the 3 projects and must be completed before the learners move on to the second part of the training, the conversation simulation. The participants are asked to complete their first conversation simulation as soon as possible after the real-time MI training and are prompted to use the platform throughout the study period.

**Figure 1:**
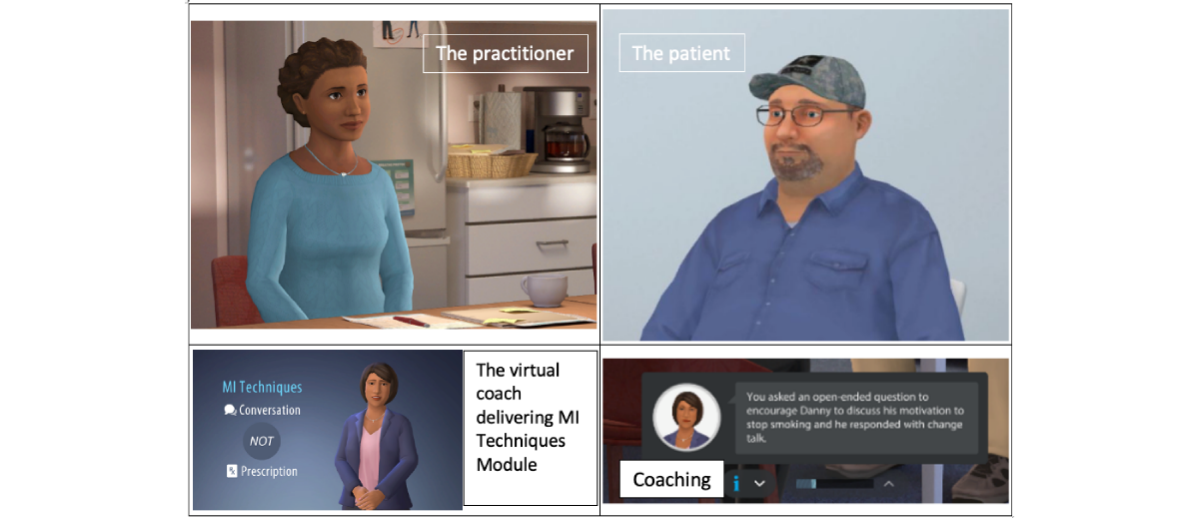
The avatar characters. MI: motivational interviewing.

Each study has 1 distinct conversation simulation that addresses the project’s respective behavioral target. All conversation simulations involve 3 characters (Figure 1). The avatar practitioner character is played by the study MI learner and discusses weight loss (ACHIEVE-D), smoking cessation (IMPACT), or cholesterol reduction (RHYTHM) with an avatar patient. The avatar patient has speech that is coded to be generated automatically; when the character stops speaking, there are 3 to 4 choices presented to the learner representing options that are more or less effective when using the guiding style of MI. Table 2 shows the learner response choices for the avatar weight loss simulation.

The avatar patient, the character that is not playable or maneuverable by the practitioner, is modeled after an atypical patient with SMI in a community mental health setting. Depending on the study, the avatar patient is obese, smokes tobacco, or has high cholesterol. The avatar patient responds to the practitioner in the way a typical client might respond as the practitioner selects one of the previous MI-consistent or non-MI directive choices. The third avatar character functions as a virtual coach who both narrates the didactic module and, when a response choice is selected by the practitioner, provides on-screen text guidance that either affirms the learner’s use of MI or, as needed, provides corrective suggestions to the learner as they engage in the respective conversations about weight loss, smoking cessation, or cholesterol reduction. Learners have the opportunity to undo their selection and pick a different option to see how such a change impacts the conversation flow.

**Figure 2:**
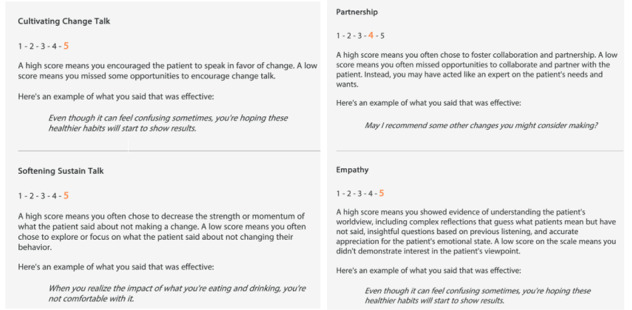
The performance dashboard.

The avatar platform includes several feedback features to assist learners in improving their MI skills. An on-screen meter registers and reinforces practitioners’ ability to cultivate change talk, a core skill that is essential in an MI interaction [7]. For example, if the practitioner says, “You’re aware of the health effects of smoking and they worry you enough to consider quitting” and the client responds, “Yea, I think about it...I used to smoke right after a shower but now I try to go longer...,” then the meter would indicate movement toward cultivating change talk. The simulations also include occasional thought bubbles; that is, a text appears above the avatar patient’s head and allows the learners to read the patient’s unspoken thoughts from the most recent exchange. This feature is most useful when the patient responds with silence, one-word answers, or if they appear hesitant to voice confusion over what is being discussed. As the learners move through the simulation, the platform keeps a tally of each practitioner’s choice; after completing the full simulation, there is a performance dashboard available to the learner on their use of MI spirit and skills. Learners are able to see their scores on the relational components of the MI spirit (partnership and empathy); adherence to the technical aspects of the approach (cultivating change talk and softening sustain talk); and their use of specific core MI skills, questions, and reflections (Figure 2). The participants are encouraged to practice their MI skills with the avatar at least monthly throughout the study.

**Table 2:** Avatar weight loss simulation learner response choices.

Client statement and practitioner choice options		Practitioner strategy	Effective use of MI^a^ approach?
***...I’m trying (to lose weight) and nothing’s happening!***			
	*Why do you think you can’t lose weight?*	Question—confrontational	No
	*You’re frustrated that this is so difficult.*	Simple reflection of sustain talk	Somewhat
	*You’re frustrated and want to do something that helps you lose weight.*	Complex reflection—shifting focus	Yes
***I get so tired climbing up all those stairs, my knee flaring’ up the whole way, that I just kind of eat whatever I want once I’m up there.***			
	*It sounds like taking those stairs would be a great way to lose weight.*	Persuading	No
	*So, all those stairs are difficult for you.*	Simple reflection of sustain talk	Somewhat
	*Even when you’re tired or frustrated, you’re still doing what needs to be done. And it makes you realize that you wish you’d eat healthier.*	Complex reflection	Yes

^a^MI: motivational interviewing.

##### Development: The Avatar Characters

Early in the development process, members of the ALACRITY team who were the most familiar with the community mental health program environment provided Kognito with pictures of the study sites and descriptions of practitioners and patients seen at the sites. After a few iterations (eg, adjusting characters’ skin tone, patient affect, and voice speed), the project’s 3 avatar characters were created. The virtual characters were brought to life with animation, lip-synced speech, and the use of facial and hand gestures to demonstrate their nonverbal emotional responses. The images of the players are the same for each of the studies. The topics of conversation and the names and mannerisms of the virtual patient characters vary by study behavioral focus. The patient avatars were modeled after clients with well-controlled depression (ACHIEVE-D), schizophrenia (smoking cessation), and bipolar disorder (CVD risk reduction) who were seen at the community mental health programs.

##### Development: The Didactic Module

Kognito’s existing MI Techniques module formed the foundation for the product’s final didactic training; the first author modified the content to reflect the most current constructs in MI [7]. Clinician and client language tailored to each study’s target behavior was incorporated so that the practitioners would find the learning module more relatable. For example, to indicate that the patient was referring to changing their smoking- or weight-related behaviors, we included examples of the patient’s change talk statements such as “I need to cut back” or “I’d like to be able to move around more easily.”

##### Development: The Conversation Simulations

To inform the script for each conversation, subject matter experts from the ALACRITY team met with Kognito. Coders then created branching scripts for how the conversations would progress as learners made choices that demonstrated both their use of MI and their options not being aligned with the patient-centered method. Practitioner choices that demonstrated the use of non-MI–adherent strategies, for example, confronting the patient’s behavior of soda drinking when their weight goes up, were carefully crafted; the MINT member authors (ATD and SM) revised the scripts designed to demonstrate MI skills (eg, asking an open-ended question to learn what the patient knows about lowering cholesterol before giving information) to more closely model effective practitioner responses. Finally, based on our experience during previous clinical trials focused on these CVD risk behaviors [8-11,41], the same authors modified the avatar patient language to better align with more predictable patient responses to each of the practitioner’s choices.

##### The Feedback Mechanisms: On-Screen Coaching

Kognito’s platform includes several feedback mechanisms for which ATD and SM provided content expertise. On-screen coaching tips from the virtual coach are available to practitioners as they move through each simulation. For example, if the learner selects the best conversation or target choice, such as, *“*Well...cutting down smoking, even a little, shows that you’re really serious about making changes for your health,” the coaching text affirms their effort and identifies the specific MI skill used: “You affirmed specific efforts Danny has made to quit smoking and he told you that in addition to his health, he values spending time with family.” If an ineffective choice is selected, such as “So, you know how difficult it is to quit,” the coach names the non-MI–adherent tactic and suggests the better or target choice: “By bringing up challenges, you missed an opportunity to affirm the efforts he’s already been making. You might have said, ‘You were able to cut out your morning cigarette even though you live with people who smoke.’”

##### The Feedback Mechanisms: The Performance Dashboard

The performance dashboard was designed to help learners know how well they use MI in their simulations. Study MI experts worked with Kognito to modify their existing automated scoring system so that it was better aligned with up-to-date MI training and feedback guidance. This was a multistep and iterative process. First, a careful review of the conversation scripts allowed the study team and Kognito to identify learner pitfalls. A pitfall by definition is when a practitioner selects a conversation strategy that is not likely to be effective when guiding the patient toward the desired behavior change (eg, if a practitioner chooses to ask an information-gathering question when a better response would be to provide a reflection). MI-nonadherent behaviors, such as persuading a patient to take a medication or confronting their lack of willingness to make a change, as well as reflecting on patient sustain talk, missing an opportunity to reflect on change talk, or failing to empathize with the patient, are coded as pitfalls. The team also identified examples of conversation targets for the learner. The targets are defined as the practitioner’s conversation choices that are the most consistent with MI in guiding the patient avatar toward the desired behavior change (eg, asking evocative questions, affirming the patient’s effort, and seeking collaboration before offering advice).

The study team was interested in having the automated performance feedback align with what is measured by the Motivational Interviewing Treatment Integrity Tool (MITI; version 4.2.1) [42]. MITI is a validated coding system that is used to assess MI skills and provide feedback to learners and is widely accepted as a treatment integrity measure for clinical trials involving MI. The tool has the following 2 components: global scores and behavior counts. Global scores capture the rater’s global impression or “gestalt” of the entire interview on 5-point Likert scales for the following 4 dimensions: Cultivating Change Talk, Softening Sustain Talk, Partnership, and Empathy. Because Artificial Intelligence is unable to characterize the “gestalt of a conversation,” the study team worked with Kognito to create separate matrices to classify the scoring for each of the MITI global domains. On the basis of the number of targets and pitfalls possible in each conversation type, we determined what combination of pitfalls and targets would comprise the grading criteria for each of the Likert scale scores (1-5). We decided to weight equally hitting the target and avoiding a pitfall. For example, if the domain of Partnership had a total possibility of 4 targets and 5 pitfalls during the weight loss conversation simulation, a *1* on the Likert scale would be assigned if the practitioner missed all the targets and hit every pitfall. A *5* on the Likert scale would be assigned if the practitioner avoided all the pitfalls and hit every target. We used this same mapping process to identify Likert score categories for each global domain for all the 3 conversation types.

MITI behavior counts are tallied instances of 10 specific practitioner behaviors including those that are categorized as MI-adherent (eg, emphasize autonomy, seek collaboration, and affirm) and MI-nonadherent (eg, confront and persuade). The human coder’s task is to simply count each instance of the clinician’s behavior during the segment being reviewed. The final step in the development of the automated scoring system for this study was to label each practitioner’s conversation choice as one of the 5 learner behaviors measured (eg, question, reflection, affirm, persuade, and confront); the automated system as currently developed was not built to provide counts for the 5 additional behaviors measured by MITI (ie, providing complex reflections, persuading with permission, giving information, seeking collaboration, and emphasizing autonomy).

### Evaluation of Training Components

#### Data Collection

Data on the measures as described in the *Measures* section will be obtained from the study practitioners before their MI training and will be collected via telephone, videoconference, or through REDCap (Research Electronic Data Capture; Vanderbilt University) or paper questionnaires. The participants will be compensated for their time to complete all data collection measures; no other financial compensation for participating in the training or implementing the study will be provided. We will use the web-based REDCap survey system or deliver paper questionnaires upon request. We will collect follow-up measures at 3 months and the end of study (at 6 months for ACHIEVE-D) and at 3 months, 6 months, and end of the study (12 months for IMPACT and RHYTHM). Continuing medical education credits will be provided to IMPACT practitioners upon the completion of 3 avatar simulations. Table 3 summarizes data collection items.

**Table 3:** Study data collection.

Measure		Time point			
		Baseline (all studies)	3 months (all studies)	6 months (all studies)	12 months (IMPACT and RHYTHM)
**Demographic characteristics**					
	Age	✓			
	Gender	✓			
	Race or ethnicity	✓			
	Length of time at program	✓			
	Role in program	✓			
	Years at clinic	✓			
	Previous formal MI^a^ training	✓			
**Beliefs about MI questionnaire**					
	A 7-question survey assessing the extent to which each person agrees with statements about MI	✓	✓	✓	✓
**Importance and confidence of using MI**					
	A 6-question survey assessing the importance and confidence each person has to deliver MI	✓	✓	✓	✓
**MITI^b^ tool**					
	Validated tool used to measure fidelity to MI from standardized actor interviews.	✓	✓	✓	✓
**Avatar MI performance measurements**					
	From use of MI techniques in simulated web-based conversations	✓	✓	✓	✓
**Perceived usefulness of avatar training questionnaire**					
	A 6-question survey assessing the extent to which each person agrees with statements about the usefulness of the asynchronous avatar training			✓^c^	✓
**Interviews**					
	Semistructured interviews using a standard protocol that will elicit participants’ perceptions of real-time and avatar MI training			✓^c^	✓

^a^MI: motivational interviewing.

^b^MITI: Motivational Interviewing Treatment Integrity.

^c^Applicable to ACHIEVE-D study only.

#### Measures

We adapted the *Beliefs about Motivational Interviewing* questionnaire [43] to assess participants’ beliefs about MI. The original scale asks participants to react to 7 statements about MI on a 5-point Likert scale from “Strongly Disagree” to “Strongly Agree.” We included the following two statements in our modified version: (1) “There is strong empirical support for motivational interviewing” and (2) “I am effectively implementing motivational interviewing.”

We created a 12-item questionnaire, *Importance and Confidence to Use MI.* The participants are asked about their perspectives on the importance of, and their self-reported confidence to use, MI skills. Example questions include, “Please rate how important is it for you to use motivational interviewing” and “Please rate how confident you are in your ability to use motivational interviewing to elicit behavior change in your patient.” Responses are chosen on a 5-point Likert scale from “Not at all” to “Extremely.”

We will use the MITI tool to measure clinician fidelity to MI from audiotaped standardized patient (SP) interviews [44-46]. Correlations between practitioners’ adherence to MI with real patients and SPs are moderate to large on most indexes of performance [47]. Practitioners will conduct interviews with SP actors playing the role of patients with SMI. SP actors will talk as if they are interested in either weight loss, smoking cessation, or CVD management according to the pilot project their practitioner interviewer is implementing. MITI scores will be determined by blinded, independent coders who were trained by the tool’s creators.

A study staff member blinded to other survey measures will extract data on practitioners’ automated scores from the performance dashboard at each data collection time point as another measure of adherence to MI.

Given the novelty of the avatar training, we will also assess participants’ perceived usefulness of the avatar platform. Perceived usefulness is a key construct in the literature on technology adoption and use and refers to the extent to which a person believes that using the technology system will enhance their job performance. We adapted the Technology Acceptance Model questionnaire [48,49] to include 6 questions about the perceived usefulness of the avatar on participants’ role in the study. Responses are on a 5-point Likert scale presented as 1 (“Strongly Disagree”) to 5 (“Strongly Agree”). Example items include “I found the avatar to be useful in my role...” and “Using the avatar enhanced my effectiveness...”

We will track adherence to the following 2 training modalities: attendance at real-time training and frequency of asynchronous avatar use.

At the end of each project, we will collect qualitative data from the interviews and focus groups using a standard protocol to elicit participants’ perceptions of both the real-time and avatar MI training.

#### Analytic Plan

We will examine changes in the MITI from baseline to each subsequent time point to assess the change in practitioners’ skills in implementing MI. All SP interviews will be coded. A difference of 1 on a 5-point scale between pre- and posttraining scores for MITI global scores will be considered a clinically significant change in MI skills proficiency and will be used for statistical testing as the relevant effect size [42].

We will use a random 20-minute segment from the audiotaped SP interviews for coding as recommended by the creators of the MITI tool [42]. We will use a random calculator to ensure that the sampling of interviews that are >20 minutes is truly random so that proper inferences about the use of MI can be drawn.

We will examine changes in practitioners’ automated scores from completed avatar simulations from baseline to each follow-up time point.

We will also compare the associations of the MITI scores from the SP interviews with the Kognito-automated performance feedback metrics from a conversation simulation completed during the same time as the data collection window.

We will examine changes in participants’ beliefs about and the importance of using MI and their confidence to use its components with their patients.

Qualitative data from interviews and focus groups will be audio recorded and transcribed. Using thematic analysis [50], 2 research team members will identify meaningful segments within the responses and assign codes. We will then group these codes into themes, which will be examined by the research team.

## Results

The ALACRITY Center was funded in August 2018. Recruitment for the studies started in February 2021. We have enrolled 127 participants. We anticipate the projects will be completed in Spring 2023 and anticipate that the results will be reported in late 2023 or 2024.

## Discussion

### Overview

The treatment of CVD risk factors is a public health priority for persons with SMI who experience symptoms disproportionally owing to these chronic health conditions. In community mental health practices where patients regularly receive their care, providing physical health interventions using an MI approach has untapped potential [4]. However, practitioners in these facilities already have excessive demands on their time, a challenge exacerbated by staff shortages and turnover, and they may not have the time, energy, or organizational support to learn how to implement patient-centered CVD risk reduction interventions [18,19]. To overcome some of the barriers to wide-scale adoption of MI in these contexts, we have adapted the traditional MI training format and created brief videoconference workshops supplemented by postworkshop avatar-based practice simulations to prepare the practitioners to deliver 3 evidence-based health interventions in community mental health settings. We hope that these formats will be acceptable and lead to an uptake and improvement of practitioners’ patient-centered skills.

### Strengths

There are several strengths in this study. We created a tailored training that has shorter duration than traditional training as a way to expose busy practitioners to MI. Brief trainings (<8 hours) are more time efficient and have been shown to make some difference in physician consultation behavior [51]. We adapted the real-time training to be delivered remotely in shorter bouts of time. We aim to identify whether the brief trainings provided are adequate to prepare real-world clinicians to perform the EBP and subsequently assess their skill acquisition with a validated measure.

Although technology-based virtual reality training methods have been used in medical and other health professionals’ education, the use of avatars and other computer programs that provide interactive learning opportunities is not as common in behavioral health skills training [25,26,30,52-54]. Virtual platforms are a relatively new, potentially promising mode for training in MI and allow busy practitioners to learn the patient-centered counseling approach from expert trainers without having to travel to an off-site location. We incorporated 3 novel avatar conversation simulations tailored for practitioners to practice and receive feedback on their use of MI in simulated discussions about weight loss, smoking cessation, and cholesterol management. We will assess their acceptability to a wide range of practitioners at the end of each project.

We describe the study’s training content and development of the avatar simulations as well as 2 methods of measuring adherence to MI and assessing change in practice that results from the trainings provided. Research has shown that the immediate skill gains from a workshop training diminish within a few months and that postworkshop follow-up performance feedback with coaching is needed for practitioners to sustain or further improve MI skills [14,16,17,55]. Although our initial training is brief, the automated coaching and feedback offered during the avatar simulations should mirror the role of coaching as a way of providing follow-up training to improve learners’ skills. Simulation technology offers the capability to move beyond traditional in-person training and provide skill training through repeated practice and feedback [56].

### Limitations

One limitation of the study is that none of the training methods have a comparator condition. The pretest-posttest outcomes are used here to assess the acceptability, feasibility, and effectiveness of the 2 training methods for improving practitioners’ MI skills.

Avatar practice requires practitioners to set aside time during their busy workdays. The extra workload and responsibilities of staff during the COVID-19 pandemic may leave little time or motivation for avatar skills practice after their initial exposure to MI, thus potentially decreasing the impact of this component of the training.

Another limitation is that because of the nature of the avatar platform and how the 3 conversations simulations are built, there are limited choice options. Therefore, after a learner has used the platform a few times, they might find it redundant and be less willing to practice. The didactic module and the conversation simulations may be a great opportunity for novice learners to practice their skills; practitioners with more experience in patient-centered counseling may be less interested in using a platform that offers few multiple-choice conversation options.

To overcome the challenges of assessing MI skills in implementation settings, we are using multiple measures, namely, MITI and the automated feedback scores from the avatar simulations. The feedback feature of the avatar simulations was originally designed to assist learners in improving MI skills. The mapping approach we used to create the automated scoring system for each of the global scores may limit the precision of the measure to capture how well the clinician is using MI. In addition, because participants complete the same simulations for training and assessment purposes, scoring from the simulations may not be a valid outcome measure of their MI skills.

This study design was in the context of implementation research and does not allow us to examine the effect of the MI workshop training component alone. However, examining MI in real-world settings is an innovative aspect of the study. Although there are limitations to asking about experiences of the training over a 6- or 12-month period, our qualitative interviews at the end of the study will add detail to the quantitative results.

Although the development of our avatar conversation simulations included multiple iterations between our study experts and the developers of the platform, further advances in virtual training have become available since the study was first conceptualized. Other programs that allow for additional branching conversation scripts could be integrated into future versions of this training to better support virtual learning [57]. Virtual reality programs that create endless iterations of interactive experiences such as those offered by SIMmersion are another option to promote learning. Platforms that use natural language processing with actual recorded patient encounters also have the potential to offer feedback to learners on their MI skills and could be an addition to MI training for busy mental health practitioners [56,58].

### Conclusions

This study will help us understand the effectiveness of 2 scalable training strategies on practitioners’ use of MI and how these approaches impact practitioners’ beliefs about, the importance of, and their confidence to use MI when delivering 3 interventions focused on CVD risk reduction to patients with SMI. The degree of tailoring to specific lifestyle change targets and the amount of real-time interaction needed to garner training effects in MI are not known; this research will contribute to knowledge about the effectiveness of brief videoconferences for training community mental health practitioners in MI. Testing MI skill uptake from videoconference training sessions has not been documented in the literature; this project will be a step forward in filling this gap. The study will also provide information about the perceived usefulness of the avatar as a modality for learning MI. On the basis of our results of this study, a logical next step to determine the most effective scalable training approach would be an RCT that compares different MI training modalities and includes a comparator condition.

## Abbreviations

CVD: cardiovascular disease
EBP: evidence-based practices
IRB: international review board
MI: motivational interviewing
MINT: Motivational Interviewing Network of Trainers
MITI: Motivational Interviewing Treatment Integrity Tool
PRP: psychiatric rehabilitation program
RCT: randomized controlled trial
REDCap: Research Electronic Data Capture
SMI: serious mental illness
SP: standardized patient

## Data Availability

The data sets generated during and analyzed during this study are not publicly available because the study is ongoing but are available from the corresponding author upon reasonable request after the study is completed. Data would be deidentified and provided to researchers whose proposed use of the data has been approved, along with a signed data access agreement and appropriate international review board approval.
